# PKM2 enhances cancer invasion *via* ETS-1-dependent induction of matrix metalloproteinase in oral squamous cell carcinoma cells

**DOI:** 10.1371/journal.pone.0216661

**Published:** 2019-05-09

**Authors:** Young-Jin Park, Jue Young Kim, Doo Young Lee, Xianglan Zhang, Shadavlonjid Bazarsad, Won-Yoon Chung, Jin Kim

**Affiliations:** 1 Oral Cancer Research Institute, Yonsei University College of Dentistry, Seoul, Korea; 2 Department of Oral Pathology, Yonsei University College of Dentistry, Seoul, Korea; 3 BK21 PLUS Project, Yonsei University College of Dentistry, Seoul, Korea; 4 Yonsei University College of Medicine, Seoul, Korea; 5 Department of Pathology, Yanbian University Hospital, Yanji City, Jilin Province, China; 6 Department of Oral Biology, Yonsei University College of Dentistry, Seoul, Korea; Queen Mary University of London, UNITED KINGDOM

## Abstract

**Objectives:**

This study aimed at investigating the molecular mechanism underlying PKM2-mediated cancer invasion.

**Materials & methods:**

To optimize the investigation of PKM2-specific effects, we used two immortalized oral cell lines. The two cell lines drastically differed in PKM2 expression level, particularly in the level of nuclear PKM2, and subsequently in glucose metabolism and tumorigenicity.

**Results:**

Knockdown of PKM2 reduced not only the glucose metabolism but also the invasive activity by curtailing the expressions of matrix metalloproteinases (MMP): PKM2 could modulate MMP-9 expression by regulating ETS-1 inside the nucleus. These results were further confirmed in an oral squamous cell carcinoma (OSCC) cell line. In correspondence with *in vitro* findings, clinicopathological data from OSCC patients indicated strong association between PKM2 expression and poor survival rate. Additionally, upon analysis of public database, significant positive correlation was found between PKM2 and ETS-1 in OSCC.

**Conclusion:**

Collectively, this study unveiled the molecular mechanism underlying PKM2-mediated cancer invasion, thereby providing novel targets for therapeutics development against invasive OSCC.

## Introduction

Cancer cells rely on aerobic glycolysis with reduced oxidative phosphorylation for glucose metabolism, a phenomenon known as the Warburg Effect.[1] Regardless of oxygen availability, cancer cells are marked by enhanced glucose uptake and lactate production.[2] Accordingly, glycolysis-associated genes such as glucose transporter 1 (GLUT1), hexokinase 2 (HK2), pyruvate kinase (PK), and lactate dehydrogenase (LDH) are upregulated in multiple types of cancer.[3] They play key roles in glycolysis by affecting several crucial steps including glucose uptake and pyruvate conversion.[4,5] Pyruvate produced during glycolysis is converted to lactate, which induces acidic tumor microenvironment and facilitates the motility and metastasis of cancer cells.[6] In particular, M2 isoform of pyruvate kinase (PKM2) has received increasing attention for its capacity to function as a protein kinase in addition to its original role as a glycolytic enzyme that regulates the rate-limiting final step of glycolysis.[7]

PKM2 differs from the M1 isoform of pyruvate kinase (PKM1) in a single exon.[7] While PKM1 can only function as a constitutively active tetrameric pyruvate kinase, PKM2 can switch between tetrameric and dimeric forms to alternate between pyruvate kinase and protein kinase.[7] As a dimeric protein kinase, PKM2 can localize to the cell nucleus, activating the transcription of multiple genes, which ultimately leads to tumor progression.[8]

Functioning as a transcriptional coactivator, nuclear PKM2 phosphorylates relevant factors to induce epigenetic changes and subsequent gene transcription. This, in turn, leads to cell cycle progression and cellular proliferation.[1,8,9] In addition, nuclear translocation of PKM2 is required for PKM2 autoregulation as well as glycolytic gene expression, to promote EGFR-dependent Warburg Effect and tumorigenesis.[10] Despite these findings on PKM2’s function as a protein kinase, identification of specific mediators between PKM2 and cancer invasion has remained elusive. Thus, this study aimed to unravel the molecular mechanism underlying PKM2-mediated cancer invasion.

For this study, two immortalized human oral keratinocyte (IHOK) cell lines with different PKM2 expression levels were chosen. These IHOKs had been transfected with high risk human papilloma virus (hr-HPV) oncogenes, for hr-HPV infection has been acknowledged as the main cause of oral squamous cell carcinoma (OSCC) and the overall incidence of OSCC associated with hr-HPV infection has gradually been increasing.[11]

Given the importance of hr-HPV infection in OSCC, we had previously established HPV16 E6/E7-transfected IHOK.[12] With the two IHOK cell lines that drastically differed in the level of PKM2, we investigated the role of PKM2 in carcinogenesis and invasion. The results obtained with IHOKs were further confirmed with OSCC cell lines. In addition, to corroborate *in vitro* findings, we conducted an *in vivo* study using OSCC patient samples and analyzed four microarray datasets obtained from the public database.

Collectively, this study showed that ETS-1, once activated by PKM2, enhances invasiveness by regulating MMP. With the excavation of molecular mechanism underlying PKM2-mediated invasion, this study will likely contribute to novel therapeutics development for the treatment of invasive OSCC.

## Materials and methods

### Cell culture

Generation of IHOK has previously been described.[12] Detailed descriptions of culture media for IHOK, human normal gingival fibroblasts (hNOF), hTERT-transfected immortalized human gingival fibroblasts (hTERT-hNOF), YD10B, CaSki, and SiHa can be found in the supplementary data. All cells were cultured at 37°C in humidified atmosphere with 5% CO_2_. All cell lines used in this study were authenticated by short-tandem repeat analysis in September 2016 at Korean Cell Line Bank. To confirm the absence of mycoplasma contamination, RT-PCR was applied.[13]

### Immunofluorescence

IHOKs were seeded in a 4-well chamber slide. Cells were fixed, permeabilized, and rinsed with PBS. Then, the cells were labeled with corresponding antibodies. Nuclei were stained with 10 μg/ml diamidinophenylindole, visualized, and photographed using confocal microscopy. Detailed description of the procedure can be found in the supplementary data.

### Reverse Transcription-Polymerase Chain Reaction (RT-PCR) and real-time PCR

Details on PCR procedures and restriction enzyme digestion can be found in the supplementary data.

### Western blotting

Details on western blot procedure can be found in the supplementary data.

### Glucose uptake assay

Cells (1 × 10^4^) were seeded in a 96-well plate for one day prior to measuring glucose uptake. After 24 h, cells were allowed to grow in glucose-free DMEM medium (Gibco BRL, #11966–025) for additional 4 h. Glucose uptake was measured using glucose uptake cell-based assay kit (Cayman Chemical, #600470), and the results were standardized by cell numbers.

### Lactate assay

Cells (1.2 × 10^5^) were cultured in serum-free medium for 48 h before collecting the conditioned medium for analysis. Lactate concentration in the collected medium was measured using Lactate Assay Kit (Biovision, #K607-100). Lactate production was standardized by cell numbers.

### Proteomic analysis

Proteins extracted from each IHOK cell line were analyzed by quantitative differential proteome analysis using 2-dimensional gel electrophoresis (2-DE) and matrix-assisted laser desorption/ionization–time-of-flight (MALDI–TOF/MS; Yonsei Proteome Research Center, South Korea).

### siRNA-mediated knockdown of gene expression

Cells were seeded one day prior to siRNA transfection in 60 mm dish and were allowed to grow up to 50% confluency. On the day of siRNA transfection, cells were transfected with siRNA in Opti-MEM (Gibco BRL, #31985–070), using Lipofectamine RNAiMax reagent (Invitrogen, #13778–075) according to the manufacturer’s instructions. Transfection efficiencies were analyzed 48 h post-transfection. The sequences of siRNAs are listed in S2 Table.[14] For knockdown of total PKM expression, sitPKM targeted the sequence common to both PKM1 and PKM2 mRNAs. For specific knockdown of PKM2, siPKM2 targeted the sequence specific to PKM2 mRNA.

### Mouse orthotopic xenograft model

Animal studies were approved by the animal ethics committee of Yonsei University College of Dentistry (2011–0067). BALB/c male mice (16 ± 2 g, 4 weeks of age) were purchased from Central Lab. Animal Inc. (South Korea). Five mice were housed per cage in a standard polycarbonate cage with temperature controlled at 22 ± 2°C and relative humidity of 50 ±10%, with 12h light and 12h dark alternation. Mice were allowed free access to sterilized food and water. Each IHOK cell line (5 × 10^5^) was injected into dorsal tongues of 15 randomized mice, respectively, and the mice were monitored twice a week. After 6 weeks, the mice were anesthetized with 1.0% isoflurane inhalation and sacrificed by carbon dioxide inhalation. Every effort was made to minimize suffering. Detailed description of tongue tumor preparation and assessment can be found in the supplementary data.

### Organotypic culture

Detailed description on the preparation of fibroblast-collagen mixture can be found in the supplementary data. IHOK cells (3 × 10^5^) were seeded on top of the fibroblast-collagen mixtures. The cultures were incubated for 4 days and raised to the air-liquid interphase for 14 days. The rafts were fixed, embedded in paraffin, sectioned and stained using hematoxylin and eosin. All invaded cells were counted by light microscopy.

### Transwell invasion assay

The inserts containing 8-μm pore in 24-well transwell plates (Corning incorporated-Life Science, #353097) were coated with Type I collagen (45 μg/30 μl/well) (Nitta Gelatin, #637–00653) and hardened for 24 h. Cells (2 × 10^4^) were placed in the upper inserts coated with type I collagen. Culture medium containing 1% FBS was placed in the lower wells. After 48 h, the cells that penetrated through the pores of inserts were fixed, stained with 0.25% crystal violet, and counted by light microscopy (Olympus, Japan). The number of invaded cells was divided by the number of total cells for normalization.

### Gelatin zymography

For detection of gelatinolytic activity, conditioned medium was loaded on SDS-polyacrylamide gel copolymerized with gelatin for electrophoresis, rinsed and incubated with buffers, then stained and destained with coomassie blue. Detailed procedure can be found in the supplementary data.

### Patient samples and construction of tissue microarray (TMA)

TMA constructed from 167 OSCC patients’ tissue sections was examined for PKM2 expression. All procedures were approved by the Institutional Review Board of Yonsei University College of Dentistry in advance (2-2011-0044). For this study, verbal informed consents were obtained from all patients. TMA block construction was performed as previously described.[15] Tissue specimens were punched out from the donor blocks using a 3mm biopsy punch (Miltex, Germany), and were planted in a recipient block. The recipient block was constructed using agar. Clinicopathological characteristics of patients were shown in Table 1.

**Table 1:** Clinicopathological characteristics of 167 oral squamous cell carcinoma patients.

Clinicopathologic variables	Number of patients (%)
*Total cases*	167
*Age (y)*	61 (26–85)
Median age (range)	
<61	76 (45.5)
≥61	91 (54.5)
*Gender*	
Male	115 (68.9)
Female	52 (31.1)
*T stage*	
T1	17 (10.2)
T2	42 (25.1)
T3	8 (4.8)
T4	100 (59.9)
*LN metastasis*	
N0	101 (60.5)
N1	26 (15.6)
N2	39 (23.4)
N3	1 (0.60)
*Histological grade*	
WD	40 (24.0)
MD	98 (58.7)
PD	29 (17.4)

### Immunohistochemical staining

Organotypic culture tissue was also examined for cytokeratin expression. Detailed description of the procedure and antibodies used can be found in the supplementary data. Interpretation of PKM2 protein expression in OSCC tissue samples was performed using the weighted histoscore method.[16] Detailed description of the method can be found in the supplementary data.

### Microarray analysis

Microarray data used in this study are available in the NCBIs Gene Expression Omnibus (GEO; http://www.ncbi.nlm.nih.gov/geo/), and can be accessed through GEO Series accession number GSE37991,[17] GDS4562.[18] Normalization was performed.

### Statistical analysis

Mann-Whitney U test, log-rank test, t-test, Pearson correlation coefficient were mainly used for statistical analysis. *P* < 0.05 was considered to be statistically significant. Details on statistical analysis can be found in the supplementary data.

## Results

### IHOK-S and IHOK-P showed different morphology and EGFR expression

**Fig 1:**
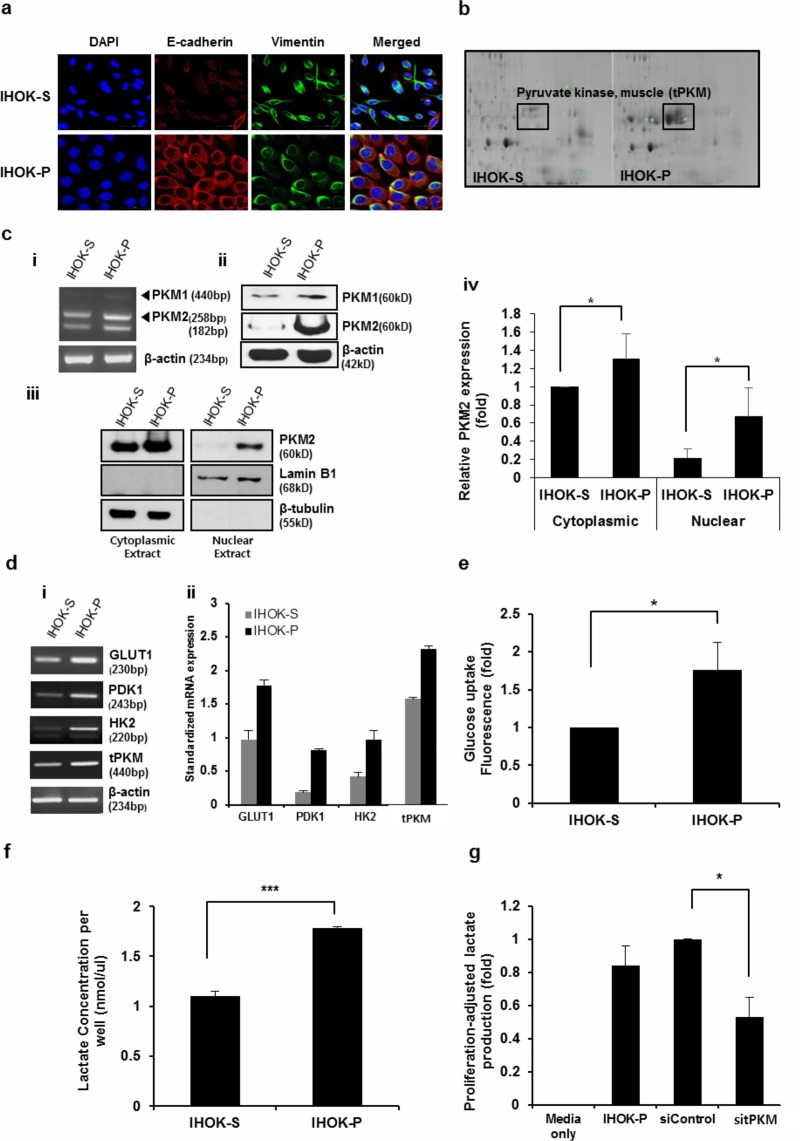
IHOK-S and IHOK-P differed in the level of total PKM (tPKM) and aerobic glycolysis. (a) In immunofluorescence microscopy, IHOK-P cells expressed lower level of Vimentin and higher level of E-cadherin compared with IHOK-S (magnification ×400; scale bar, 50 μm). (b) Proteomic analysis between IHOK-S and IHOK-P pinpointed on total PKM as a protein whose expression differed significantly between the two cell lines. (c) RNA (i) and protein (ii) expressions of PKM1 and PKM2. IHOK-P had higher PKM1 and PKM2 levels than IHOK-S in both RT-PCR and western blotting. (iii and iv) In particular, IHOK-P had much higher levels of nuclear PKM2 than IHOK-S. (d) i) mRNA expressions of GLUT1, PDK1, HK2, and total PKM were measured by RT-PCR in IHOK-S and IHOK-P. ii) Results were normalized by β-actin expression. (e and f) Glucose uptake and lactate production were analyzed. Glucose uptake and lactate production were standardized by cell numbers. The results were shown as mean ± SD (*n* = 3) and analyzed by the Mann-Whitney U test (*P < 0.05, ***P < 0.001). (g) Knockdown of total PKM (sitPKM) in IHOK-P led to reduction in lactate production, indicating that PKM facilitates aerobic glycolysis in IHOK-P. The results were shown as mean ± SD (*n* = 3), and were analyzed by the Mann-Whitney U test (*P < 0.05).

The mRNA expressions of HPV16 E6 and E7 genes were examined in IHOK cell lines (S1A Fig). Since two IHOK cell lines exhibited different morphology, each IHOK cell line was named as IHOK-S and IHOK-P. IHOK-S lacked cell-to-cell contact and displayed spindle shape. IHOK-P exhibited polygonal shape and cohered with adjacent cells. IHOK-P cells expressed lower level of Vimentin and higher level of E-cadherin than IHOK-S (Fig 1A and S1B Fig). IHOK-P also had higher expression of EGFR than IHOK-S (S1C Fig).

## IHOK-S and IHOK-P showed different levels of tPKM expression and aerobic glycolysis

Proteomic analysis revealed tPKM to be one of the most significantly upregulated proteins in IHOK-P compared with IHOK-S (Fig 1B). Consistent with these results, IHOK-P had higher mRNA and protein expressions of PKM1 and PKM2 compared with IHOK-S (Fig 1C i, ii, iii). IHOK-P had 3.1-fold higher level of nuclear PKM2 than IHOK-S (Fig 1C iv). IHOK-P had 2.3-fold higher nuclear-to-cytoplasmic PKM2 ratio than IHOK-S (S1D Fig). Expressions of genes involved in aerobic glycolysis such as GLUT1, pyruvate dehydrogenase kinase 1 (PDK1), HK2, and tPKM were also analyzed: IHOK-P had higher mRNA expressions of glycolysis-associated genes compared with IHOK-S (Fig 1D). In addition, IHOK-P had 1.8-fold higher glucose uptake and 1.6-fold higher lactate production than IHOK-S (Fig 1E and 1F). To assess the effect of tPKM on lactate production in IHOK-P, tPKM expression was knocked down using siRNA (sitPKM) that targets both PKM1 and PKM2. Depletion of tPKM led to reduction in lactate production (Fig 1G). In addition, short-term and long-term growth rates were compared between the two cell lines to check whether the difference in growth rate could affect the experimental results. IHOK-P cells exhibited higher long-term growth rate than IHOK-S (S2A and S2B Fig). However, glucose uptake and lactate assays were conducted at 24 h and 48 h, respectively, the time points that did not induce significant proliferative difference between the two cell lines. Therefore, the difference in growth rates between the two cell lines could be ignored in assessing glucose uptake and lactate assay results.

### IHOK-P had higher tumorigenicity, higher invasiveness, and higher MMP expression than IHOK-S

**Fig 2:**
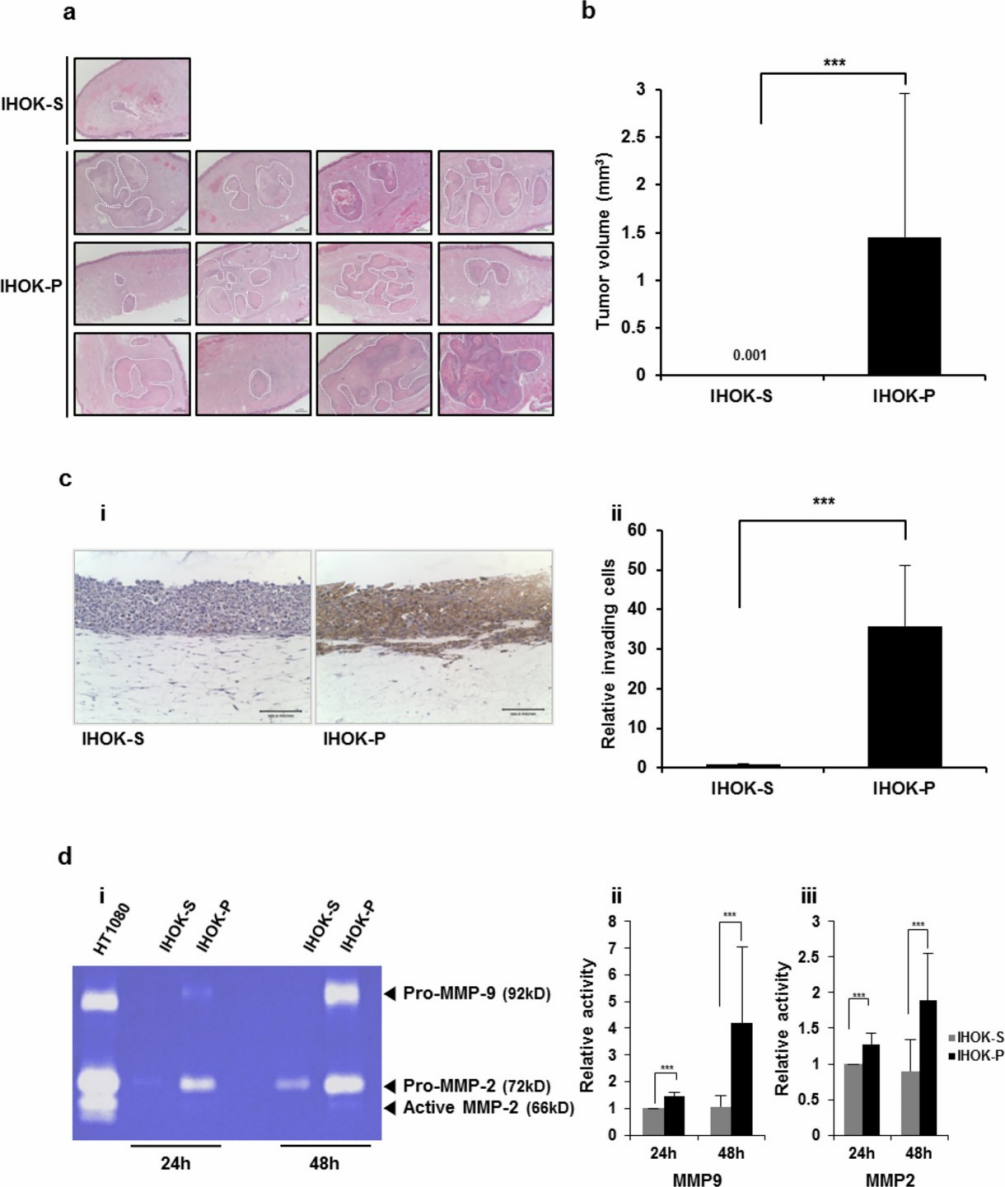
Compared with IHOK-S, IHOK-P had higher tumorigenicity, higher invasiveness, and higher MMP expression. (a) Histological sections of mice tongues injected with IHOK-S or IHOK-P cells. IHOK-S or IHOK-P cells (5 × 10^5^) were injected into the dorsal tongue of nude mouse (magnification ×40; scale bar, 100 μm). The tumor margin is indicated by a dashed line. (b) Difference in average tumor volume between mice tongues injected with IHOK-S and those injected with IHOK-P. The results were analyzed by the Mann-Whitney U test (***P < 0.001). (c) i) Invasive activity of IHOK-S and IHOK-P cells was determined by 3-dimensional organotypic culture with immunohistochemical staining for cytokeratin (magnification ×200; scale bar, 100 μm). ii) IHOK-P cells more readily invaded into dermal equivalent. All invaded cells were counted by light microscopy. The results were shown as mean ± SD (*n* = 3) and analyzed by the Mann-Whitney U test (***P < 0.001). Note that this experiment was performed to measure the invasiveness of IHOK-S and IHOK-P, not to measure the level of cytokeratin staining. Cytokeratin was not expressed in IHOK-S, and thus IHOK-S was poorly stained. (d) i) Enzymatic activities of MMP-2 and MMP-9 were measured in IHOK-S and IHOK-P using zymography. IHOK-P cells showed higher MMP-9 (ii) and MMP-2 (iii) activities compared with IHOK-S cells for both 24-hour and 48-hour incubation. HT1080 was used as a positive control. The results were shown as mean ± SD (*n* = 3) and analyzed by the Mann-Whitney U test (***P < 0.001).

To assess whether the tumorigenic potential correlates with the level of aerobic glycolysis, equal number of IHOK-S or IHOK-P cells were injected into dorsal tongue of nude mouse. Four mice injected with IHOK-S and two mice injected with IHOK-P died in the middle of experiment. The injected mice were monitored for 6 weeks and sacrificed. Only 1 out of 11 mice (9.1%) developed tumor in IHOK-S-injected group. In contrast, 12 out of 13 mice (92.3%) developed tumor in IHOK-P-injected group (S3A and S3B Fig). The tumors formed after the injection of IHOK-P were significantly larger in size than those formed by IHOK-S (Fig 2A and 2B).

Next, invasion assay was performed using IHOK-S and IHOK-P. With transwell invasion assay, we could not detect difference in invasive activity between the two cell lines, because the two cells were not equal in size and shape (S4A Fig). With 3-dimensional organotypic culture, invasive activity of IHOK-P was significantly higher than that of IHOK-S. All invaded cells were counted by light microscopy after cytokeratin staining (Fig 2C). In accordance with the results from S1B Fig, IHOK-S displayed poor epithelial differentiation as evidenced by the lack of cytokeratin expression in Fig 2C.

Then, expressions of extracellular matrix degradation enzymes were examined. IHOK-P had higher expressions of MMP-2 and MMP-9 than IHOK-S. In contrast, IHOK-P had lower expressions of tissue inhibitors of metalloproteinase (TIMP)-1, TIMP-2, and TIMP-4 than IHOK-S (S4B Fig). IHOK-P showed markedly higher MMP-2 and MMP-9 expressions in real-time PCR (S4C Fig). Zymographic analyses confirmed the enhanced activities of MMP-2 and MMP-9 in IHOK-P compared with those in IHOK-S (Fig 2D).

### PKM2 knockdown reduced the invasiveness of IHOK-P

**Fig 3:**
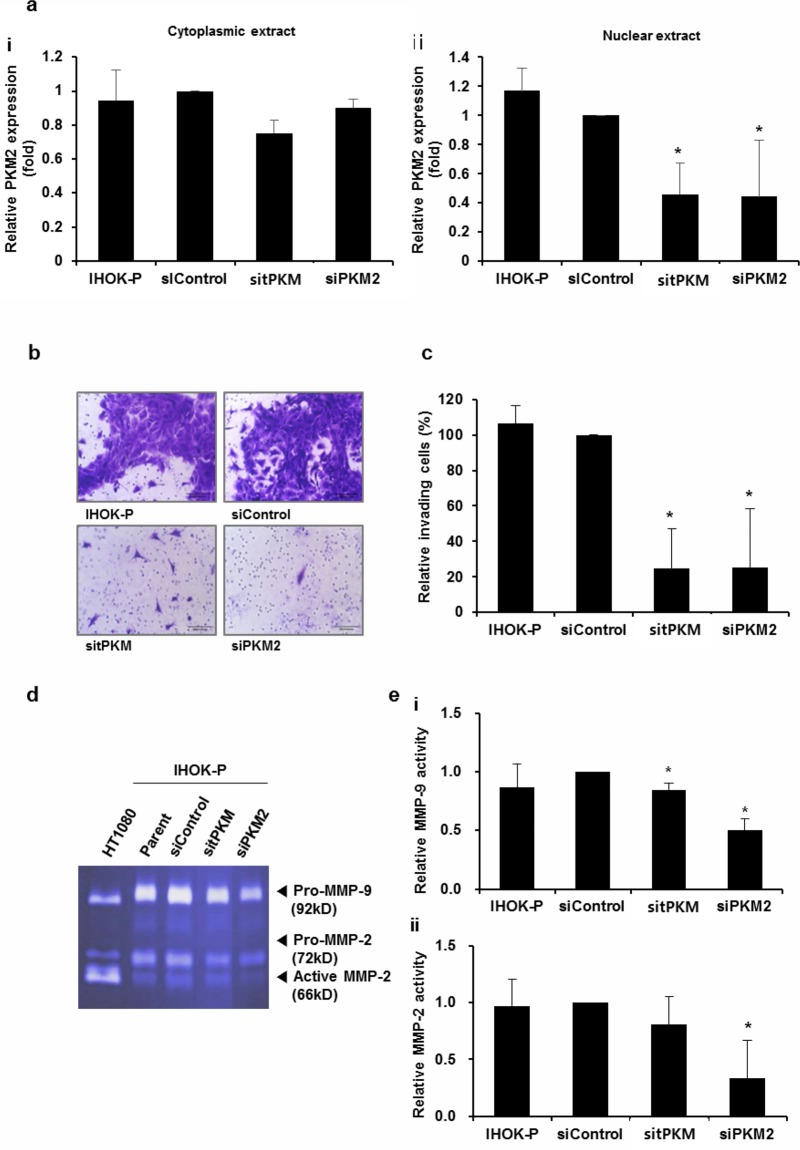
PKM2 knockdown reduced the invasiveness of IHOK-P. (a) Effects of PKM2 knockdown were separately assessed for cytoplasmic (i) and nuclear (ii) extracts following the depletion of tPKM or PKM2. The results were shown as mean ± SD (*n* = 3). (b and c) Invasive activity of IHOK-P following the depletion of tPKM or PKM2 (magnification ×200; scale bar, 100 μm). Knockdown of tPKM or PKM2 in IHOK-P led to reduction in invasion. The number of invaded cells was divided by the total number of cells for normalization and presented as % invading cells. The results were shown as mean ± SD (*n* = 3) and analyzed by the Mann-Whitney U test (*P < 0.05). (d and e) MMP-2 and MMP-9 activities were examined in IHOK-P by gelatin zymography after the specific depletion of tPKM and PKM2, respectively. HT1080 was used as a positive control for the activities of MMP-2 and MMP-9. The activities of MMP-2 and MMP-9 decreased significantly following the depletion of PKM2. The results were shown as mean ± SD (*n* = 3) and analyzed by the Mann-Whitney U test (*P < 0.05).

To test whether the upregulation of PKM2 is responsible for higher invasiveness in IHOK, siRNA against PKM2 was introduced into IHOK-P. When the effects of PKM2 knockdown were separately assessed for cytoplasmic and nuclear PKM2 proteins, more drastic decrease was observed for nuclear PKM2 (Fig 3A). Significant reduction in invasiveness was observed in IHOK-P following PKM2 depletion (Fig 3B and 3C). Zymographic analyses confirmed that the activities of MMP-2 and MMP-9 were significantly reduced after PKM2 knockdown in IHOK-P (Fig 3D and 3E), indicating that PKM2 plays an important role in regulating MMP.

### PKM2 knockdown reduced the invasiveness *via* downregulation of ETS-1

**Fig 4:**
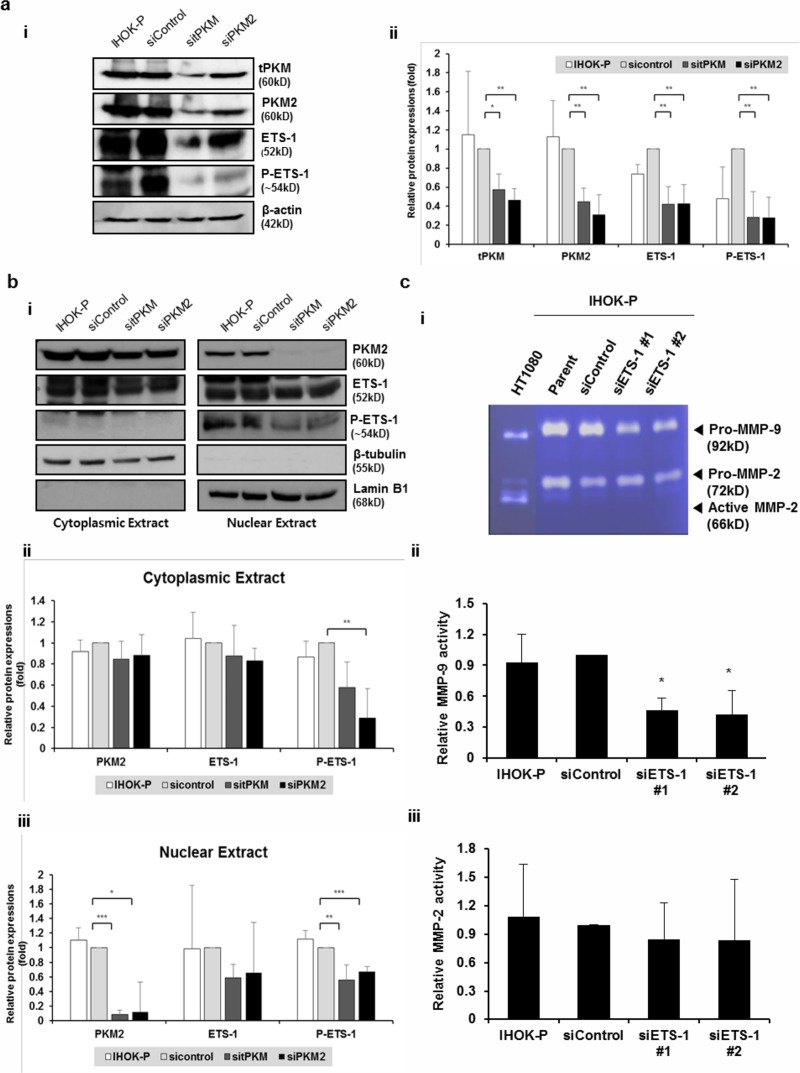
PKM2 modulates ETS-1 expression and phosphorylation in IHOK-P. (a) (i) Expressions of ETS-1 and phosphorylated ETS-1 were significantly reduced at the protein level following tPKM or PKM2 knockdown. (ii) The results were shown as mean ± SD (*n* = 3) and analyzed by the Mann-Whitney U test (*P < 0.05, **P < 0.01). (b) (i) Significant reduction in PKM2 and phospho-ETS-1 levels was observed in the nuclear compartment, when the effects of PKM2 knockdown were separately assessed for cytoplasmic and nuclear proteins. Phospho-ETS-1 was not detected in the cytoplasmic compartment. (ii and iii) The results were shown as mean ± SD (*n* = 3) and analyzed by the Mann-Whitney U test (*P < 0.05, **P < 0.01, ***P < 0.001). (c) (i) MMP-2 and MMP-9 activities were measured by gelatin zymography in IHOK-P following ETS-1 depletion. (ii) Activity of MMP-9 decreased significantly following the depletion of ETS-1. The results were shown as mean ± SD (*n* = 3) and analyzed by the Mann-Whitney U test (*P < 0.05).

To better understand the molecular mechanism by which PKM2 regulates MMPs, we measured the expressions of molecules known as the transcriptional regulators of MMPs. Compared with IHOK-S, IHOK-P had higher expressions of ETS-1, SP1, ATF2, cJUN, and cFOS. Among these, mRNA expression of ETS-1 was significantly reduced after PKM2 knockdown (S5 Fig). Protein expressions of ETS-1 and phosphorylated ETS-1 at threonine 38 were also reduced after the depletion of PKM2 in IHOK-P (Fig 4A). The reduction of PKM2 and the subsequent decrease of phospho-ETS-1 were more prominent in the nuclear extract than in the cytoplasmic extract after PKM2 knockdown. In fact, phospho-ETS-1 proteins were only detected in the nuclear extract of IHOK-P (Fig 4B).

To confirm that ETS-1 is indeed involved in regulating MMP expression in IHOK, we measured the activities of MMP-2 and MMP-9 after knocking down ETS-1 in IHOK-P. Although both MMP-2 and MMP-9 expressions were significantly reduced after PKM2 knockdown as shown in Fig 3D and 3E, only MMP-9 activity was significantly reduced after ETS-1 depletion (Fig 4C).

**Fig 5:**
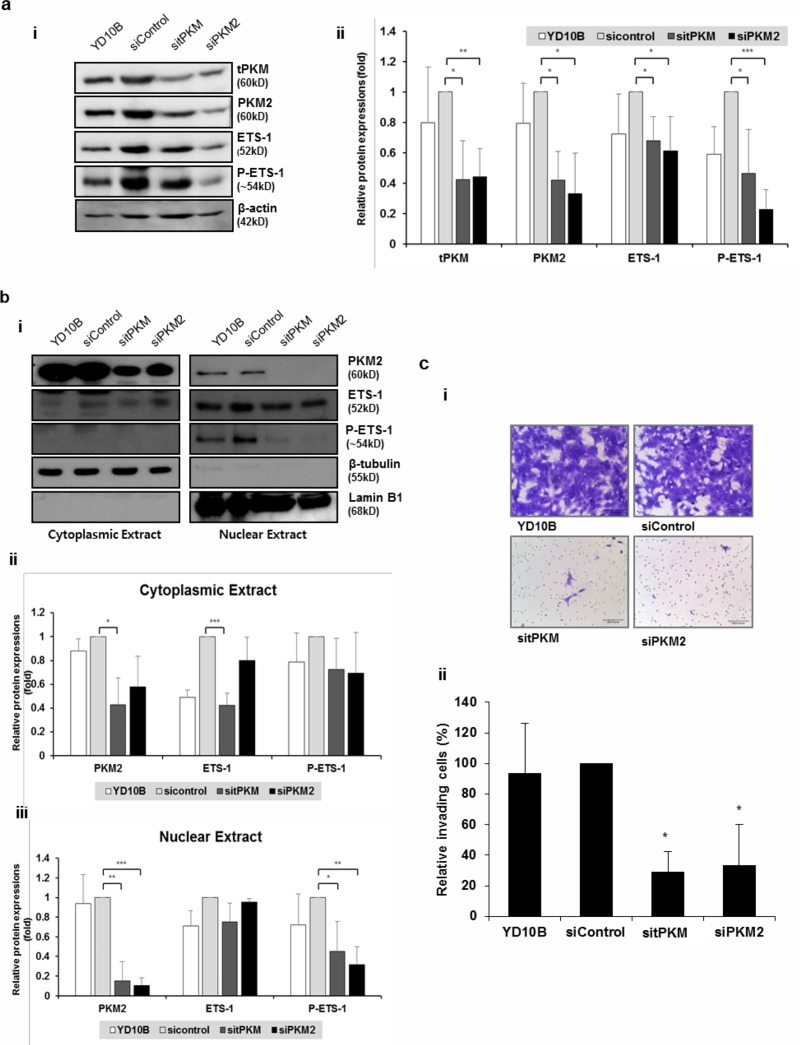
PKM2 modulates ETS-1 phosphorylation in oral squamous carcinoma cells. (a) (i) Protein levels of ETS-1 and phosphorylated ETS-1 were measured after PKM2 knockdown in YD10B. (ii) The results were shown as mean ± SD (*n* = 3) and analyzed by the Mann-Whitney U test (*P < 0.05, **P < 0.01, ***P < 0.001). (b) (i) Effects of PKM2 knockdown were separately assessed for cytoplasmic and nuclear proteins. (ii and iii) The results were shown as mean ± SD (*n* = 3) and analyzed by the Mann-Whitney U test (*P < 0.05, **P < 0.01, ***P < 0.001). (c) (i) Knocking down tPKM or PKM2 in YD10B led to reduction in invasive activity (magnification ×200; scale bar, 100 μm). (ii) The number of invaded cells was divided by the number of total cells for normalization and presented as % invading cells. The results were shown as mean ± SD (*n* = 3), and were analyzed by the Mann-Whitney U test (*P < 0.05).

To corroborate these findings in cancer cells, PKM2 was knocked down in YD10B, an OSCC cell line. In accordance with the results obtained in IHOK-P, both ETS-1 and phosphorylated ETS-1 levels were significantly reduced by PKM2 knockdown in YD10B cells (Fig 5A). When the effects of PKM2 knockdown were separately assessed for cytoplasmic and nuclear proteins, PKM2 depletion and subsequent reduction in phospho-ETS-1 were observed only in the nuclear extract of YD10B cells (Fig 5B). PKM2 knockdown resulted in reduced invasion of YD10B cells (Fig 5C).

### PKM2 level was negatively correlated with the survival rate for OSCC patients

**Fig 6:**
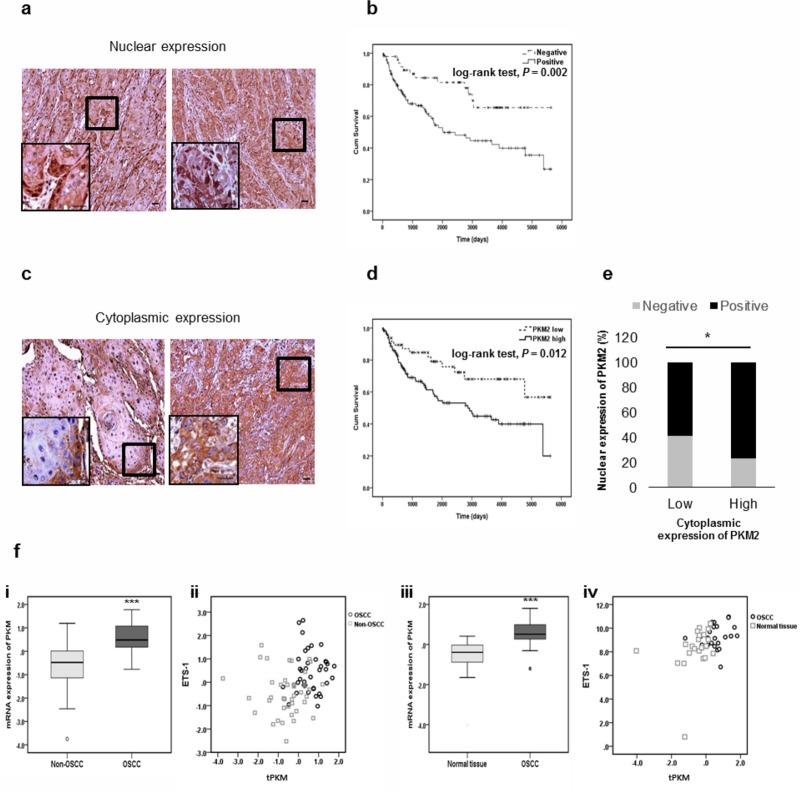
PKM2 level is negatively correlated with the survival rate of OSCC patients. (a) Immunohistochemical staining with anti-PKM2 antibodies was performed on 167 OSCC TMA. The micrographs shown in this figure are representative of two nuclear positive groups (magnification ×200; scale bar, 20 μm). Nuclear expression patterns were observed at the invasive front (left) or scattered in invasive cell nests (right). Higher magnification (×1000) view was presented in the inset micrograph. (b) Overall survival of 167 patients with OSCC classified into negative- or positive- nuclear expression. Patients with positive expression of nuclear PKM2 had poorer survival rates than patients with negative expression. The results were analyzed by the log-rank test (*P* = 0.002). (c) The micrographs shown in this figure are representative of cytoplasmic-low (left) and cytoplasmic-high (right) groups (magnification ×200; scale bar, 20 μm). Higher magnification (×1000) view was presented in the inset micrograph. (d) Overall survival of 167 patients with OSCC classified into low- or high- cytoplasmic expression. Patients with high expression had poorer survival rates than patients with low expression. The results were analyzed by the log-rank test (*P* = 0.012). (e) Significant association was found between cytoplasmic and nuclear PKM2 expression by chi-square test (P = 0.024). (f) (i) Expression level of tPKM was significantly increased (***P < 0.001) in OSCC tumors (*n* = 40) compared with the adjacent non-OSCC tissues (*n* = 40). (ii) Correlation between tPKM expression and ETS-1 expression in OSCC (*n* = 40) and non-OSCC tissues (*n* = 40). Weak correlation was found (Pearson correlation = 0.250, *P* = 0.025). (iii) Expression level of tPKM was significantly increased (***P < 0.001) in OSCC tissues (*n* = 24) compared with the adjacent normal oral tissues (*n* = 24). (iv) Correlation between tPKM expression and ETS-1 expression in OSCC (*n* = 23) and normal oral tissues (*n* = 23). Moderate correlation was found (Pearson correlation = 0.407, *P* = 0.005). In scatter plots, black dots denote tumor samples, while grey dots denote control samples.

To confirm *in vitro* findings, immunohistochemistry was performed using PKM2 antibodies in 167 OSCC patient samples (Table 1). Expression of cytoplasmic PKM2 was detected in all tumor cells of OSCC patients except for 3 patients, showing no statistically significant prognostic difference between positive and negative expression. For nuclear PKM2, positive expression was observed mainly at the invasive fronts or scattered in the invasive cell nests of OSCC (Fig 6A), and was detected in 119 OSCC patients (71.3%). Significant difference in survival rate was observed between patients with positive (Median survival duration: 974 days) and negative (Median survival duration: 2585 days) nuclear PKM2 expression (Fig 6B).

PKM2 expression was also classified into high and low expression. For cytoplasmic PKM2, diffuse high expression was detected in 119 OSCC patients (71.3%) (Fig 6C). Significant difference in survival rate was observed between patients with high (Median survival duration: 981 days) and low (Median survival duration: 1902 days) expression (Fig 6D). Similarly, significant difference in survival rate was observed between patients with high (Median survival duration: 842 days) and low (Median survival duration: 1755 days) nuclear PKM2 expression (S6 Fig). Significant association was observed between the cytoplasmic and nuclear expression of PKM2 (Fig 6E).

### Expressions of PKM2 and ETS-1 were positively correlated in OSCC public datasets

The analyses of four microarray datasets obtained from the public database (GEO: Gene Expression Omnibus) were conducted to confirm our results. Expression patterns of tPKM selected from two independent OSCC datasets are shown in Fig 6F. tPKM expression was higher in cancer groups than their respective control groups for OSCC (Fig 6F i and iii). Intriguingly, in the correlation analysis between the expressions of tPKM and ETS-1, the OSCC datasets showed positive relationship (Fig 6F ii and iv).

## Discussion

The aim of the current study was to address the veiled roles of PKM2 in cancer invasion by elucidating the underlying mechanism in OSCC. Two immortalized oral keratinocyte cell lines, IHOK-S and IHOK-P, were chosen for this study: the two cell lines exhibited different morphological and molecular characteristics despite sharing the same integration sites for HPV16 E6/E7 genes.[19] Proteomic analysis between the two cell lines pinpointed on tPKM as one of the most significantly upregulated proteins in IHOK-P compared with IHOK-S. In addition, IHOK-P had much higher EGFR expression than IHOK-S, as shown in S1C Fig, suggesting the activation of EGFR-PKM2 axis in IHOK-P.

Nuclear translocation of dimeric PKM2 is promoted both by tumor-specific characteristics and tumor microenvironment such as excessive EGFR activation and hypoxia.[10,20] To illustrate, EGFR activation leads to ERK2-PKM2 binding and phosphorylation of PKM2 at S37, which facilitates PIN1-mediated cis-trans isomerization of PKM2. This isomerization exposes the nuclear localization signal of PKM2 to importin α5, thereby promoting the translocation of PKM2 into the nuclear compartment.[10,21] In accordance with these findings, higher EGFR expression was associated with enhanced nuclear translocation of PKM2 in IHOK in the present study.

The levels of PKM2, both nuclear and cytoplasmic, were higher in IHOK-P than in IHOK-S, contributing to high tumorigenic and invasive potential of IHOK-P in the present study. Based on previous studies, nuclear PKM2 functions as a histone kinase and upregulates the expression of c-Myc and cyclin D1, thereby facilitating cell cycle progression and aerobic glycolysis.[21] Thus, IHOK-P showed higher long-term growth rate than IHOK-S, at least partly contributing to higher *in vivo* tumorigenicity. As glycolytic PKM2 offers selective advantages to cancer cells by facilitating nucleotide synthesis and rapid generation of ATP,[22,23] the higher level of cytoplasmic PKM2 would have facilitated glycolysis in IHOK-P, supplying the cells with diverse substrates required for proliferation. Taken together, it is likely that nuclear and cytoplasmic PKM2 work in concert to promote tumor progression, although the relative importance of roles that PKM2 plays in each compartment might differ depending on the specific type of cancer.

This study focused on unraveling the detailed mechanism of PKM2 in promoting cancer invasion. Depletion of nuclear PKM2 led to significant reduction in MMP-2 and MMP-9 expressions and consequently, in the invasiveness of IHOK-P. An important step in MMP regulation is transcription: ETS-1 has been reported to be overexpressed in a variety of human cancers and plays a major role in transactivation of several protease genes involved in matrix degradation such as MMP-1, MMP-2, and MMP-9.[24] Capable of enhancing the invasive potential of cancer cells both *in vitro* and *in vivo*,[25] ETS-1 posed itself as a potent mediator that might bridge upregulated PKM2 and enhanced invasion in IHOK-P.

In our study, knockdown of nuclear PKM2 led to decreased expressions of ETS-1, phosphorylated ETS-1, and subsequently MMP-9 in IHOK-P, indicating a regulatory role of nuclear PKM2 in induction of MMP-9 *via* ETS-1. Although both MMP-2 and MMP-9 activities were reduced after nuclear PKM2 knockdown, ETS-1 knockdown did not result in significantly reduced MMP-2 activity, indicating that nuclear PKM2 might modulate MMP-2 via pathways that do not necessarily involve ETS-1. Effect of PKM2 knockdown on ETS-1 expression and phosphorylation was further confirmed in YD10B, an OSCC cell line. Similar to IHOK, the effects of PKM2 knockdown in YD10B were more evident for nuclear compartment proteins, with drastically reduced nuclear PKM2 and nuclear phospho-ETS-1 levels after the knockdown, eventuating in reduced invasive activity. All of the aforementioned findings were attained in both PKM2 knockdown and total PKM knockdown cells, indicating that PKM2 rather than PKM1 is really the driving force responsible for the invasiveness of IHOK-P and OSCC cells. Specific association between PKM1 and invasiveness was not investigated due to PKM1’s inability to enter nucleus and function as a protein kinase.

To corroborate our *in vitro* data on PKM2, an *in vivo* study was conducted using OSCC patient samples and corresponding results were obtained: higher PKM2 expression, both nuclear and cytoplasmic, resulted in poorer survival rates. A recent study emphasized the association between PKM2 expression and aggressive clinicopathological features in OSCC, supporting our results.[26]

In addition, we screened public database to assess tPKM’s potential to function as a robust biomarker in various types of cancer. Transcriptome profiling was collected from non-tumor and tumor tissue samples for OSCC. As results, tPKM expression was negatively correlated with survival rate of cancer patients in all of the samples analyzed. In addition, positive correlation between tPKM and ETS-1 was found in the OSCC datasets. Taken together, PKM2 facilitates cancer invasion *via* ETS-1 pathway in OSCC.

To conclude, our study offered the evidence that PKM2 plays a crucial role in invasion of OSCC cells *via* ETS-1-mediated regulation of MMP-9. We hope to further unravel the entangled connections between abnormal glucose metabolism and cancer progression to facilitate development of novel therapeutics against invasive cancers.

## Supporting information

S1 Fig Two IHOK cell lines exhibited different molecular characteristics. (a) mRNA expressions of HPV-16 E6 and E7 were measured by RT-PCR. IHOK-KGM, SiHa, and CaSki were used as positive controls for HPV-16 E6 and E7 expression. β-actin was used as a loading control. (b) Expressions of genes associated with keratinocyte differentiation and mesenchymal marker genes were measured in IHOK-S and IHOK-P by RT-PCR and Western blot. IHOK-P cells expressed lower level of Vimentin and higher level of Involucrin. GAPDH was used as a loading control in RT-PCR. β-actin was used as a loading control in Western blot. Normal fibroblast was used as a positive control for Vimentin. (c) Difference in EGFR expression between IHOK-P and IHOK-S as measured by Western blot. Levels of EGFR and phosphorylated EGFR were higher in IHOK-P than in IHOK-S. (d) Nuclear-to-cytoplasmic PKM2 ratio was measured in IHOK-S and IHOK-P by Western blot.
(TIF)

S2 Fig Two IHOK cell lines differed in long-term proliferative activity. (a) The number of proliferated cells was counted 1 day, 2 days, and 3 days after cell seeding. The results were shown as mean ± SD (*n* = 3), and were analyzed by the Mann-Whitney U test (*P < 0.05). (b) IHOK-P had 1.36 times higher long-term proliferative activity than IHOK-S when the proliferation was measured for more than 60 days.
(TIF)

S3 Fig Compared with IHOK-S, IHOK-P had higher tumorigenicity. (a) Gross view of mice tongues injected with IHOK-S (Lower) and IHOK-P (Upper) cells. The approximate tumor margin is indicated by a dashed line. (b) Only one mouse (9.1%) developed tumor in the IHOK-S-injected group. In contrast, 12 of 13 mice (92.3%) developed large tongue tumors in the IHOK-P-injected group.
(TIF)

S4 Fig Compared with IHOK-S, IHOK-P had higher MMP expression. (a) Invasive activity of IHOK-S and IHOK-P cells was evaluated by transwell-invasion assay. There was no significant difference in invasive activity between IHOK-S and IHOK-P (i and ii). (b) (i) IHOK-P cells expressed higher levels of MMP-2 and MMP-9 compared with IHOK-S cells in RT-PCR. GAPDH was used as a loading control. (ii) IHOK-P cells expressed lower levels of TIMP-1, TIMP-2, and TIMP-4 than IHOK-S in RT-PCR. β-actin was used as a loading control. (c) Expression levels of different types of MMPs in IHOK-S and IHOK-P. IHOK-P showed much higher expressions of MMP-2 and MMP-9 compared with IHOK-S in real-time PCR.
(TIF)

S5 Fig PKM2 modulates ETS-1 transcription in IHOK-P. (a) Levels of transcription factors that regulate MMP expression were assessed in IHOK-S and IHOK-P (i and ii). Levels of transcription factors that regulate MMP expression were assessed following tPKM or PKM2 knockdown in IHOK-P (iii and iv).
(TIF)

S6 Fig Nuclear PKM2 level is negatively correlated with the survival rate of OSCC patients. Overall survival of 167 patients with OSCC classified into low- or high- nuclear PKM2 expression. Significant difference in survival rate was observed between patients with high and low nuclear PKM2 expression. The results were analyzed by the log-rank test (*P* = 0.010).
(TIF)

S1 Table Sequences of primers used for PCR and RT-PCR. (DOCX)

S2 Table Sequences of siRNAs used in this study. (DOCX)

S1 Materials and Methods (DOCX)
