# Supplementary figures and images for: PKM2 enhances cancer invasion via ETS-1-dependent induction of matrix metalloproteinase in oral squamous cell carcinoma cells
Source: PLoS One. 2019 May 9;14(5):e0216661. doi: 10.1371/journal.pone.0216661 (PMC6508653; doi:10.1371/journal.pone.0216661)

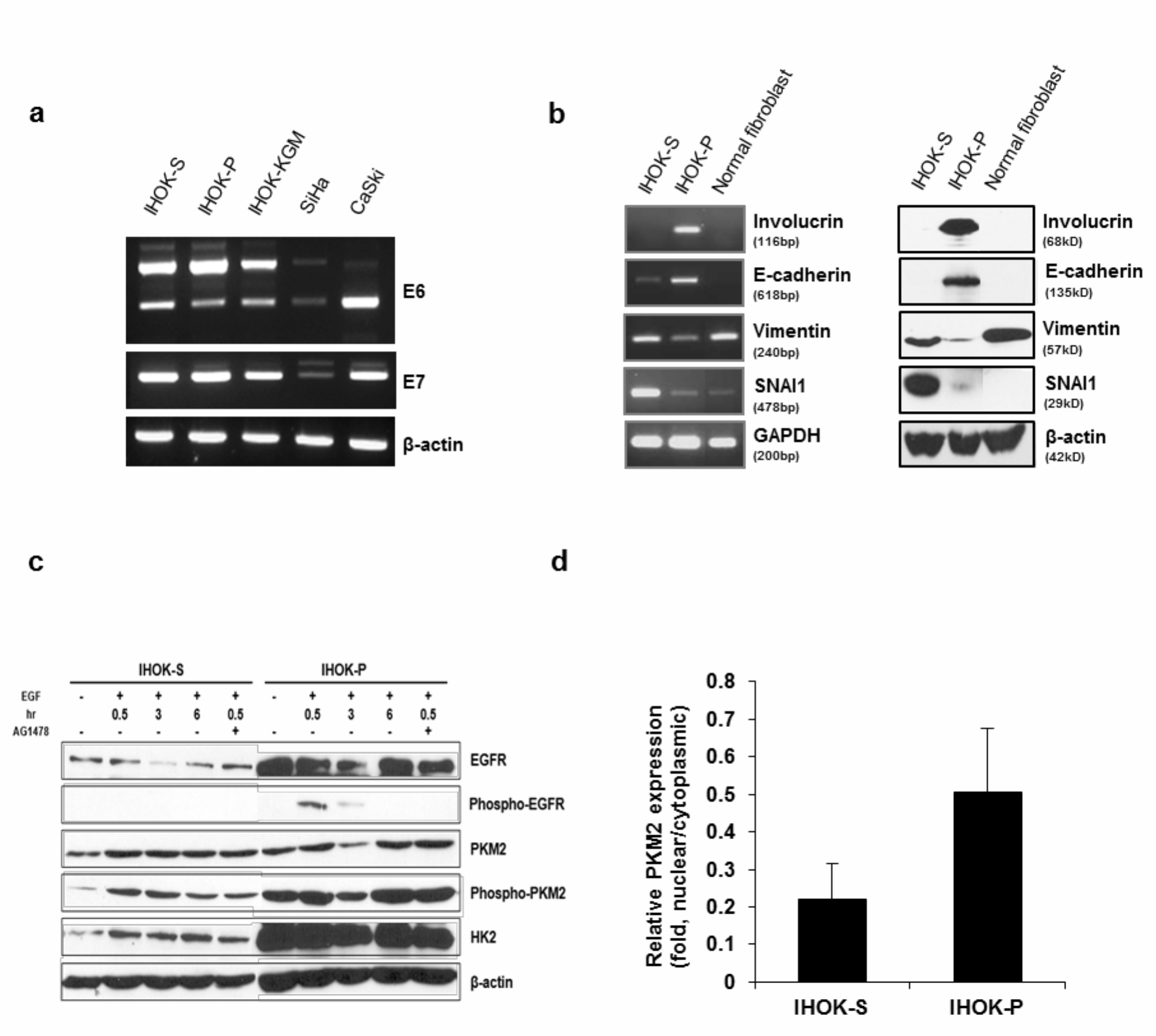

Supplement: S1 Fig — (a) mRNA expressions of HPV-16 E6 and E7 were measured by RT-PCR. IHOK-KGM, SiHa, and CaSki were used as positive controls for HPV-16 E6 and E7 expression. β-actin was used as a loading control. (b) Expressions of genes associated with keratinocyte differentiation and mesenchymal marker genes were measured in IHOK-S and IHOK-P by RT-PCR and Western blot. IHOK-P cells expressed lower level of Vimentin and higher level of Involucrin. GAPDH was used as a loading control in RT-PCR. β-actin was used as a loading control in Western blot. Normal fibroblast was used as a positive control for Vimentin. (c) Difference in EGFR expression between IHOK-P and IHOK-S as measured by Western blot. Levels of EGFR and phosphorylated EGFR were higher in IHOK-P than in IHOK-S. (d) Nuclear-to-cytoplasmic PKM2 ratio was measured in IHOK-S and IHOK-P by Western blot. (TIF) [file pone.0216661.s001.tif]

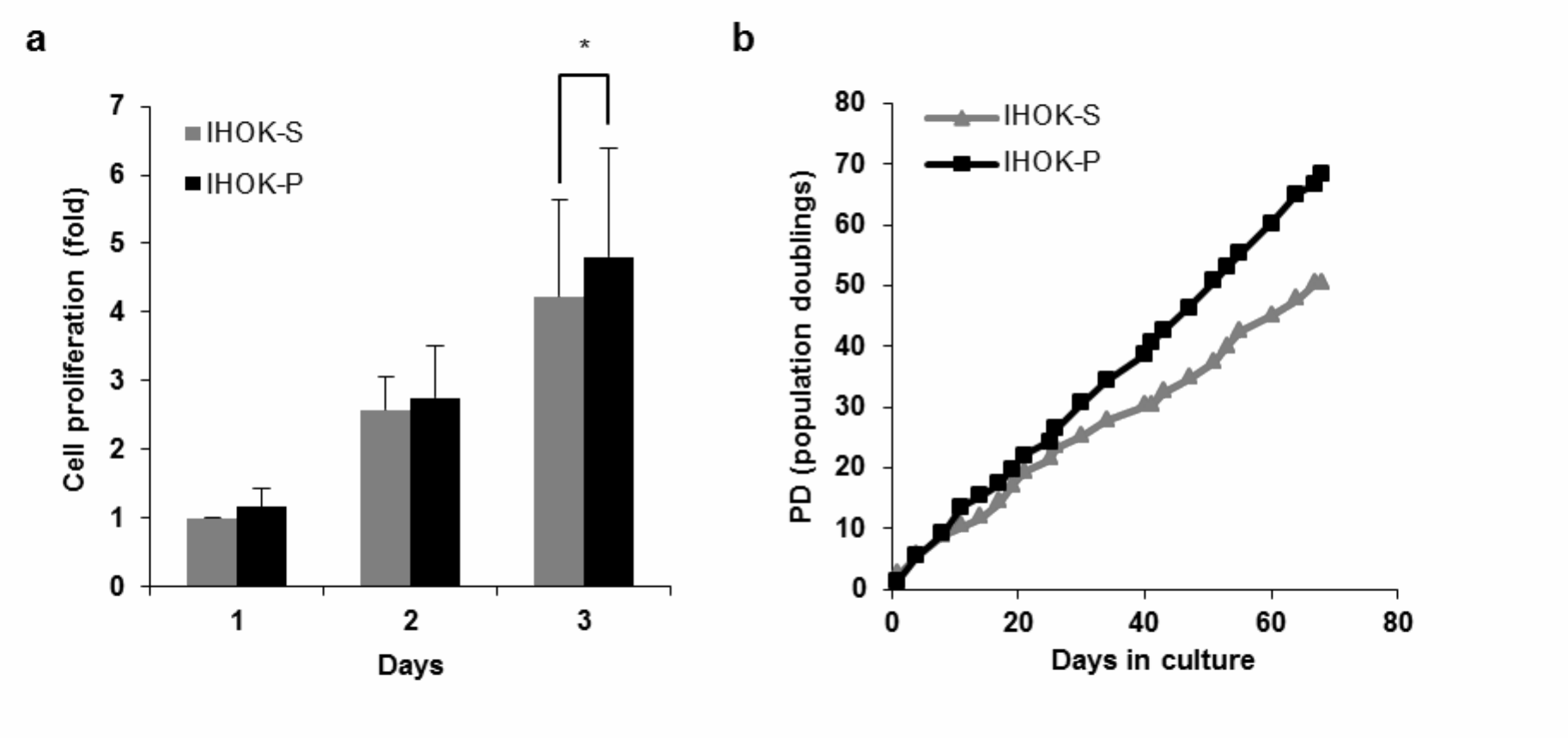

Supplement: S2 Fig — (a) The number of proliferated cells was counted 1 day, 2 days, and 3 days after cell seeding. The results were shown as mean ± SD (n = 3), and were analyzed by the Mann-Whitney U test (*P < 0.05). (b) IHOK-P had 1.36 times higher long-term proliferative activity than IHOK-S when the proliferation was measured for more than 60 days. (TIF) [file pone.0216661.s002.tif]

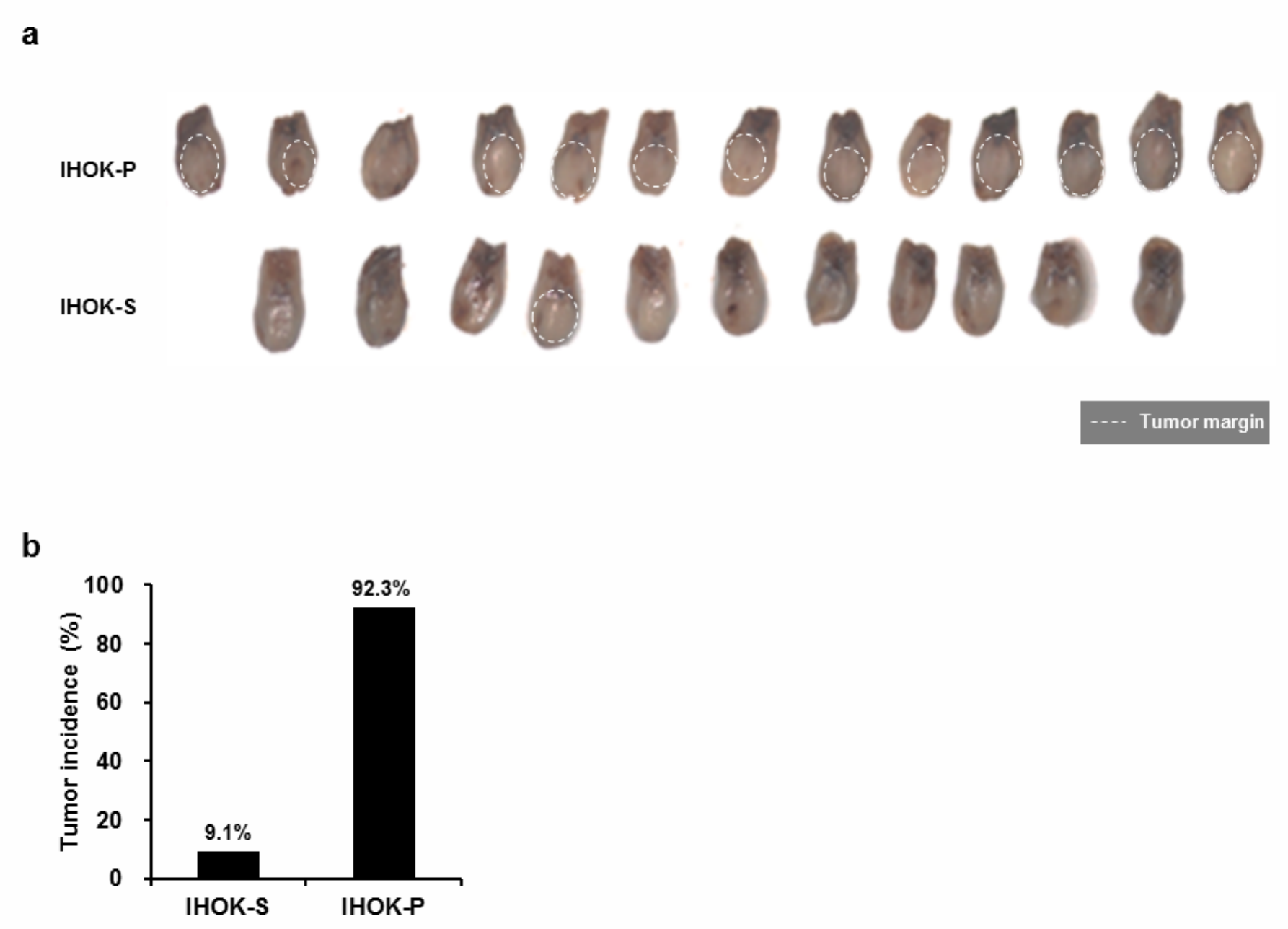

Supplement: S3 Fig — (a) Gross view of mice tongues injected with IHOK-S (Lower) and IHOK-P (Upper) cells. The approximate tumor margin is indicated by a dashed line. (b) Only one mouse (9.1%) developed tumor in the IHOK-S-injected group. In contrast, 12 of 13 mice (92.3%) developed large tongue tumors in the IHOK-P-injected group. (TIF) [file pone.0216661.s003.tif]

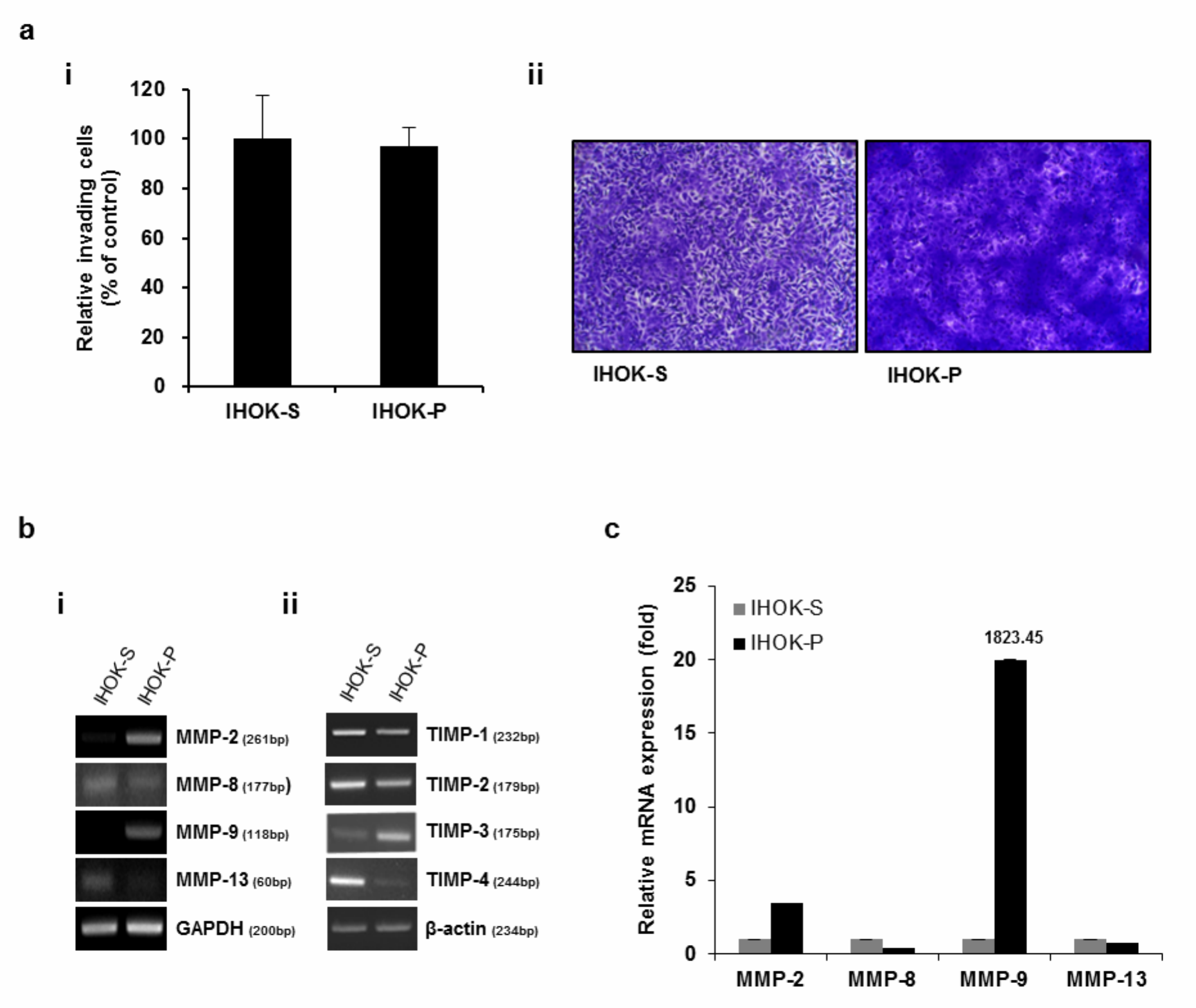

Supplement: S4 Fig — (a) Invasive activity of IHOK-S and IHOK-P cells was evaluated by transwell-invasion assay. There was no significant difference in invasive activity between IHOK-S and IHOK-P (i and ii). (b) (i) IHOK-P cells expressed higher levels of MMP-2 and MMP-9 compared with IHOK-S cells in RT-PCR. GAPDH was used as a loading control. (ii) IHOK-P cells expressed lower levels of TIMP-1, TIMP-2, and TIMP-4 than IHOK-S in RT-PCR. β-actin was used as a loading control. (c) Expression levels of different types of MMPs in IHOK-S and IHOK-P. IHOK-P showed much higher expressions of MMP-2 and MMP-9 compared with IHOK-S in real-time PCR. (TIF) [file pone.0216661.s004.tif]

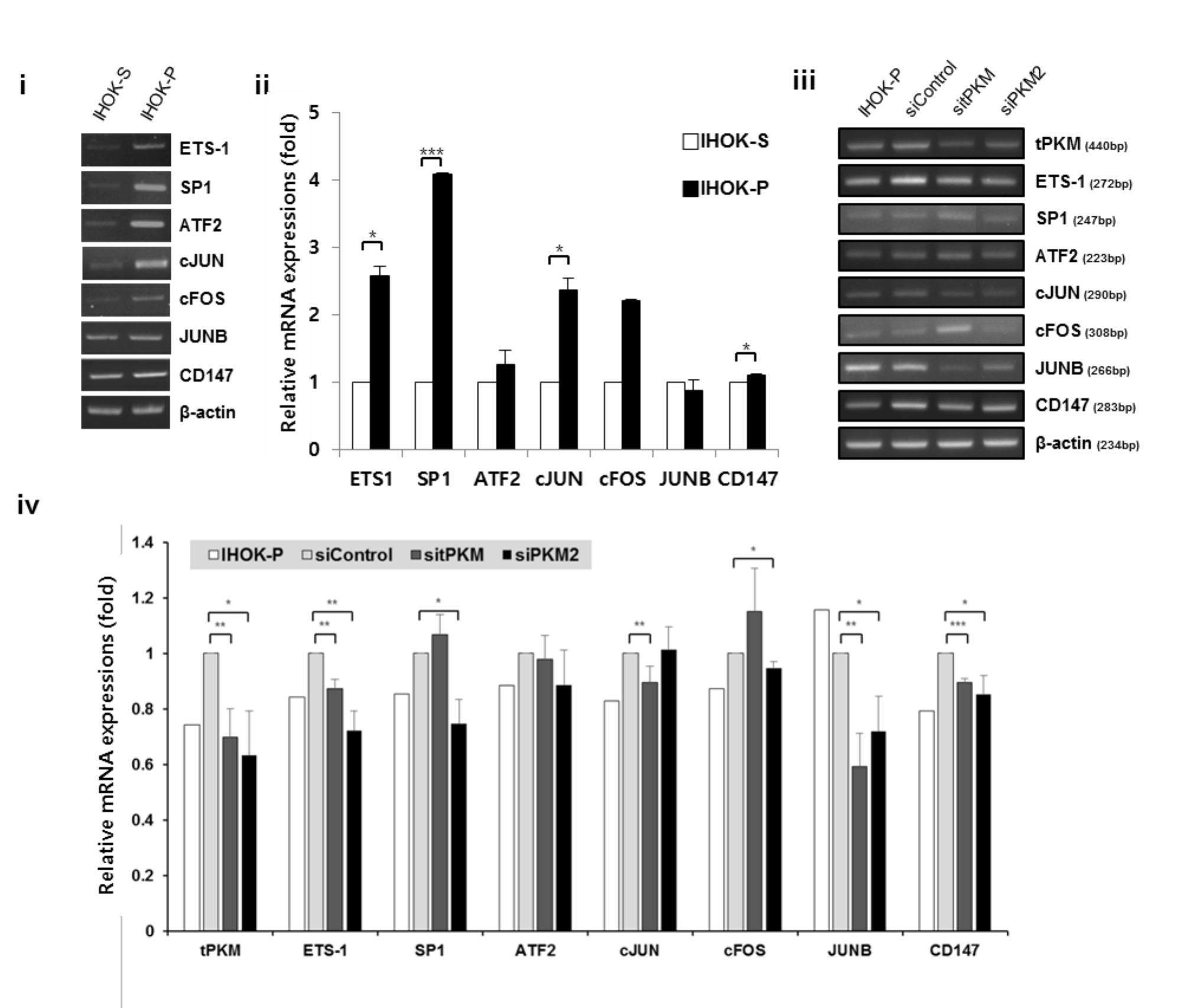

Supplement: S5 Fig — (a) Levels of transcription factors that regulate MMP expression were assessed in IHOK-S and IHOK-P (i and ii). Levels of transcription factors that regulate MMP expression were assessed following tPKM or PKM2 knockdown in IHOK-P (iii and iv). (TIF) [file pone.0216661.s005.tif]

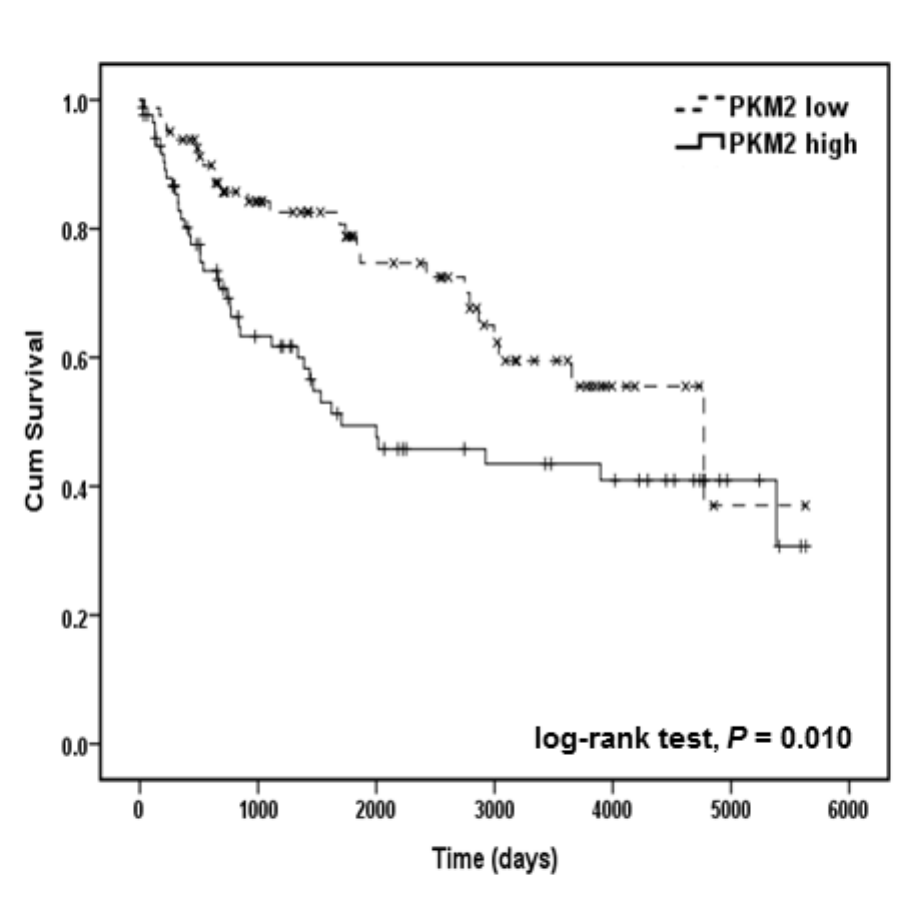

Supplement: S6 Fig — Overall survival of 167 patients with OSCC classified into low- or high- nuclear PKM2 expression. Significant difference in survival rate was observed between patients with high and low nuclear PKM2 expression. The results were analyzed by the log-rank test (P = 0.010). (TIF) [file pone.0216661.s006.tif]
