# Supplementary material for: PKM2 enhances cancer invasion via ETS-1-dependent induction of matrix metalloproteinase in oral squamous cell carcinoma cells
Source: PLoS One. 2019 May 9;14(5):e0216661. doi: 10.1371/journal.pone.0216661 (PMC6508653; doi:10.1371/journal.pone.0216661)
Supplement: S1 Table — (DOCX) [file pone.0216661.s007.docx]

| S1 Table. Sequences of primers used for PCR and RT-PCR | | |
| --- | --- | --- |
| **Gene** | **Sense sequence (5' to 3')** | **Antisense sequence (5' to 3')** |
| Involucrin | GGGTGGTTATTTATGTTTGGGTGG | GCCAGGTCCAAGACATTCAAC |
| E-cadherin | CCTGGGCAGAGTGAATTTTGAAGA | TCATTCTGATCGGTTACCGTGATC |
| Vimentin | GACAATGCGTCTCTGGCACGTCTT | TCCTCCGCCTCCTGCAGGTTCTT |
| SNAI1 | ACTACAGCGAGCTGCAGGACTC | GTGTGGCTTCGGATGGTCAT |
| GLUT1 | CTTCACTGTCGTGTCGCTGT | TGAAGAGTTCAGCCACGATG |
| PDK1 | CACGCTGGGTAATGAGGATT | ACTGCATCTGTCCCGTAACC |
| HK2 | TCTATGCCATCCCTGAGGAC | TCTCTGCCTTCCACTCCACT |
| MMP2 | AGGGCATTCAGGAGCTCTAT | CCTCGTATACCGCATCAATC |
| MMP8 | CTCACAGGGAGAGGCAGATA | TTGTAATTTGCGGAGGTGTT |
| MMP9 | TTCTCCAGAAGCAACTGTCC | CCCTCAAAGGTTTGGAATCT |
| MMP13 | CAGACAAATGTGACCCTTCC | TTGTTTCTCCTCGGAGACTG |
| MT1-MMP | GGATACCCAATGCCCATTGGCCA | CCATTGGGCATCCAGAAGAGAGC |
| TIMP1 | ACCAGAAGTCAACCAGACCA | AGCCACAAAACTGCAGGTAG |
| TIMP2 | GAAGAAGAGCCTGAACCACA | AGCCGTCACTTCTCTTGATG |
| TIMP3 | CCTTAAGCTGGAGGTCAACA | GCAGGACTTGATCTTGCAGT |
| TIMP4 | CCAACAGCCAGAAGCAGTAT | CCATAGAGCTTTCGTTCCAA |
| tPKM | AACAGCCAAAGGGGACTATC | CAATGACCACATCTCCCTTC |
| PKM1 | CGAGCCTCAAGTCACTCCAC | GTGAGCAGACCTGCCAGACT |
| PKM2 | ATTATTTGAGGAACTCCGCCGCCT | ATTCCGGGTCACAGCAATGATGG |
| ETS-1 | CAGACTTTGTTGGGGACATC | GACCGAGGGGTAGTCATTCT |
| SP1 | GCTACCTGTCAACAGCGTTT | CCCTGATGATCCACTGGTAG |
| ATF2 | CAGCTCACACAACTCCACAG | TTTCTCAGCAGGGTGACTTC |
| cJUN | GATAATCCAGTCCAGCAACG | AGTTGCTGAGGTTTGCGTAG |
| cFOS | GGAGGGAGCTGACTGATACA | GCTGATGCTCTTGACAGGTT |
| JUNB | GGCAGCTACTTTTCTGGTCA | GGTGTCACGTGGTTCATCTT |
| CD147 | TACTCCTCACCTGCTCCTTG | ACGGACTCTGACTTGCAGAC |
| GAPDH | GAAGGTGAAGGTCGGAGT | GAAGATGGTGATGGGATTTC |
| β-actin | GGACTTCGAGCAAGAGATGG | AGCACTGTGTTGGCGTACAG |
| E6 | ATGTTTCAGGACCCGCAGGAGCGA | TTACAGCTGGGTTTCTCTACGTG |
| E7 | TGTTAGATTTGCAACCAGAGACA | TTATGGTTTCTGAGAACAGATGGG |
