# Supplementary material for: PKM2 enhances cancer invasion via ETS-1-dependent induction of matrix metalloproteinase in oral squamous cell carcinoma cells
Source: PLoS One. 2019 May 9;14(5):e0216661. doi: 10.1371/journal.pone.0216661 (PMC6508653; doi:10.1371/journal.pone.0216661)
Supplement: S2 Table — (DOCX) [file pone.0216661.s008.docx]

S2 Table. Sequences of siRNAs used in this study

| **Gene** | **Sense sequence (5' to 3')** | **Antisense sequence (5' to 3')** |
| --- | --- | --- |
| sitPKM #1 | GCUGUGGCUCUAGACACUAAA | UUUAGUGUCUAGAGCCACAGC |
| sitPKM #2 | UGUCGGAGAAGGACAUCCAG | CUGGAUGUCCUUCUCCGACA |
| siPKM2 | CCAUAAUCGUCCUCACCAA | UUGGUGAGGACGAUUAUGG |
| siETS-1 #1 | CUGGUUUUGGACGACAGUA(dTdT) | UACUGUCGUCCAAAACCAG(dTdT) |
| siETS-1 #2 | GCAUAGAGAGCUACGAUAG(dTdT) | CUAUCGUAGCUCUCUAUGC(dTdT) |
