# Supplementary material for: PKM2 enhances cancer invasion via ETS-1-dependent induction of matrix metalloproteinase in oral squamous cell carcinoma cells
Source: PLoS One. 2019 May 9;14(5):e0216661. doi: 10.1371/journal.pone.0216661 (PMC6508653; doi:10.1371/journal.pone.0216661)
Supplement: S1 Materials and Methods — (DOCX) [file pone.0216661.s009.docx]

***Supplementary data for***

**PKM2 enhances cancer invasion *via* ETS-1-dependent induction of matrix metalloproteinase in oral squamous cell carcinoma cells**

Young-Jin Park ^1,2¶^, Jue Young Kim ^1,3¶^, Doo Young Lee ^1,4¶^, Xianglan Zhang ^1,5^, Shadavlonjid Bazarsad ^1,2^, Won-Yoon Chung^1,3,6^ and Jin Kim ^1,2,3*^

^1^ Oral Cancer Research Institute, Yonsei University College of Dentistry, Seoul, Korea

^2^ Department of Oral Pathology, Yonsei University College of Dentistry, Seoul, Korea

^3^ BK21 PLUS Project, Yonsei University College of Dentistry, Seoul, Korea

^4^ Yonsei University College of Medicine, Seoul, Korea

^5^ Department of Pathology, Yanbian University Hospital, Yanji City, Jilin Province, China

^6^ Department of Oral Biology, Yonsei University College of Dentistry, Seoul, Korea

* Corresponding author

E-mail: [jink@yuhs.ac](mailto:jink@yuhs.ac) (JK)

^¶^ These authors contributed equally to this work as the co-first authors.

**Total number of Supplementary figures/ tables:** 6/2

**Materials and methods**

**Cell culture**

IHOK was cultured in mixed medium that contained Dulbecco’s Modified Eagles Medium (DMEM; Gibco BRL, #12800-017) and Ham’s Nutrient Mixture-F12 (Gibco BRL, #21700-075) mixed at a ratio of 3:1 supplemented with 10% fetal bovine serum (FBS; Corning Cellgro, #35-015-CV), 1% penicillin/ streptomycin (Gibco BRL, #15140-163), 0.01 μg/ml cholera toxin (Sigma, #C-8052), 0.04 μg/ml hydrocortisone (Sigma, #H-4001), 0.5 μg/ml insulin (Sigma, #I-5500), 0.5 μg/ml apo-transferrin (Sigma, #T-2252), and 0.2 μg/ml 3’-5 triodo-1-thyronine (Sigma, #T-6397) (EF-medium). Human normal gingival fibroblasts (hNOF) and hTERT-transfected immortalized human gingival fibroblasts (hTERT-hNOF) were previously described.[1] YD10B, an OSCC cell line, was maintained in EF-medium.[2] CaSki and SiHa were cultured in RPMI 1640 medium (Gibco BRL, #22400-089) supplemented with 10% FBS and 1% penicillin/streptomycin.

**Immunofluorescence**

IHOKs were seeded in a 4-well chamber slide. Cells were fixed in 4% formaldehyde for 15 min, permeabilized in 0.5% triton X-100 solution, and rinsed three times in PBS. Then, the cells were labeled with antibodies against E-cadherin (1:100, R&D Systems, #MAB1838) or Vimentin (1:100, Cell Signaling Technology, #5741S). Secondary antibodies, goat anti-mouse Alex 594 (for E-cadherin; Invitrogen, #A-11005) and goat anti-mouse Alex 488 (for Vimentin; Invitrogen, #A-11008) 1:2000, were added and incubated for 1 h at room temperature. Then, nuclei were stained with 10 µg/ml diamidinophenylindole (DAPI; Sigma, #D8417), visualized, and photographed using confocal microscopy (LSM 700 Meta, Germany).

**Reverse Transcription-Polymerase Chain Reaction (RT-PCR) and real-time PCR**

Total cellular RNA was extracted from cells using an RNeasy Mini Kit (Qiagen, #74134) according to the manufacturer’s instructions. Complementary DNA was synthesized using Transcriptor First Strand cDNA Synthesis Kit (Roche, #4896866001) according to the manufacturer’s instructions. The oligonucleotide primers were synthesized by Macrogen (South Korea), and are listed in S1 Table. The cDNA product was amplified by PCR using an Accu Power Hot Start PCR Pre Mix (Bioneer, #K-5051) with the following conditions: 30 cycles of 30 s at 94 °C, 40 s at 58 °C or 60 °C, and 40 s at 72 °C. The amplified products were then separated on 1.0% agarose gels stained with 0.1 μg/ml ethidium bromide and photographed under UV light (Bio-Rad, USA). Expressions of PKM1 and PKM2 mRNAs were measured *via* RT-PCR followed by PstI digestion. PstI, a restriction enzyme that recognizes and cuts CTGCAG sequence, digests exon 10 (the PKM2-specific exon) but not exon 9 (the PKM1-specific exon). Undigested band on the top corresponds to PKM1, while two digested bands on the bottom correspond to PKM2.[3,4] Real-time PCR was carried out using SYBR Green I Master (Roche, #4707516001) and normalized to GAPDH. LightCycler 480 Software (Roche Applied Science, Switzerland) was used for data analysis.

**Western blotting**

Cells were lysed using cell lysis buffer (Cell Signaling Technology, #9803). To separate nuclear and cytoplasmic proteins, NE-PER Nuclear and Cytoplasmic extraction reagents (Thermo Fisher Scientfic, #78833) were used. Forty micrograms of protein were separated by SDS-polyacrylamide gel (Bio-Rad Laboratories, USA) and transferred to polyvinylidene fluoride membrane (Bio-Rad Laboratories, #1620177). The membranes were incubated with appropriate primary antibodies specific for E-cadherin (#4065), SNAI1 (#3895), vimentin (#5741), ETS1 (#6258), tPKM (#3190), PKM1 (#7067), PKM2 (#3198), Lamin B1 (#12586), β-Tubulin (#2146), epidermal growth factor receptor (EGFR) (#2232), phospho-EGFR (#2237), phospho-PKM2 (#3827) (1:1000, Cell Signaling Technology), involucrin (1:1000, Affinity BioReagents, #PA1-37934), phospho-ETS1 (1:1000, Bioworld Technology, #BS4316), EGFR (1:1000, Abcam, #ab52894), and anti-actin antibody (1:1000, Sigma, #A2066). Proteins were detected with horseradish peroxidase-conjugated anti-mouse or anti-rabbit IgG antibodies (1: 2000, Cell Signaling Technology, #7076S/#7074S) and visualized by chemiluminescence (Santa Cruz Biotechnology, #SC-2048) according to the provided protocol.

**Preparation of fibroblast-collagen mixture for organotypic culture**

Collagen mixture was prepared by mixing eight volumes of type I collagen solution (Nitta Gelatin, #637-00653), one volume of 10× reconstitution solution (0.022 g/ml NaHCO_3_, 0.0477 g/ml HEPES and 0.05 N NaOH), and one volume of 10× DMEM (Gibco BRL, #12800-017) and Ham’s Nutrient Mixture-F12 (Gibco BRL, #21700-075) mixed at a ratio of 3:1. hTERT-hNOF (1.5 × 10^5^) were mixed with 250 μl collagen mixture. The cell-collagen mixture was placed into a Mill-cell (3.0 μm pore size, 12 mm diameter; Millipore, #PITP01250) and allowed to be polymerized at 37 ºC for 24 h.

**Immunohistochemical staining**

Organotypic culture tissue was also examined for cytokeratin expression. Antibodies specific for cytokeratin AE1/AE3 (1:100, Dako, #M3515), PKM2 (1:800, Cell Signaling Technology, #4053) were applied at room temperature for 90 min. Anti-IgG antibody (1:100, R&D Systems, #MAB004) was used as a negative control. The secondary EnVision™ Rabbit/Mouse reagent (DAKO, #K5007) was then applied at room temperature for 30 min. The sections were visualized with 3, 3-diaminobenzidine tetrachloride (DAB) and counter-stained with Mayer’s haematoxylin. Interpretation of PKM2 protein expression in OSCC tissue samples was performed using the weighted histoscore method.[5] Total histoscore of cytoplasmic expression was calculated based on staining intensity and distribution. The intensity was scored as 0 (negative), 1 (light brown), 2 (brown), or 3 (dark brown). Total histoscore was calculated as follows: total histoscore = (0 × percentage of negative cells) + (1 × percentage of cells with light brown staining) + (2 × percentage of cells with brown staining) + (3 × percentage of cells with dark brown staining). For analysis, patients were subdivided into two groups: low (total histoscore 0-100) and high (101-300) expression groups. Nuclear expression was evaluated by measuring the percentage of positive cells. Low expression and high expression groups were classified based on the median.

**Mouse orthotopic xenograft model: morphologic analysis & assessment of tumor**

Tongues of the mice were fixed in 10% neutral formalin, embedded in paraffin, sectioned, and stained with hematoxylin and eosin for morphologic analysis. Tumor size was measured using the following formula: 0.5 × a × b^2^, where a and b are the long and short diameters, respectively.

**Gelatin zymography**

Cells (1 × 10^6^) were seeded in a 100 mm dish and allowed to adhere overnight. Serum-free medium was added and incubated for 24 h or 48 h. For detection of gelatinolytic activity, the conditioned medium was centrifuged and quantified for protein content. Thirty micrograms of each sample were loaded on a 8% SDS-polyacrylamide gel (Bio-Rad Laboratories, USA) copolymerized with 0.3% gelatin (Sigma, #2625). After electrophoresis, the gels were washed to remove SDS, rinsed with zymogram renaturation buffer (Bio-Rad Laboratories, #161-0765) for 2 h, and then incubated for 16 h at 37 ºC in zymogram development buffer (Bio-Rad Laboratories, #161-0766). After washing, the gels were stained for 2 h with coomassie blue and destained.

**Statistical analysis**

To determine whether there is difference between the control and experimental groups, Mann-Whitney U test (SPSS Inc, USA) was performed. The overall survival of OSCC patients was estimated with Kaplan-Meier method using the log-rank test. For microarray data analysis, t-test and Mann-Whitney U test were used between the tumor and normal samples. Pearson correlation coefficient was used to measure the strength of the linear relationship between tPKM and ETS-1 expressions. *P* < 0.05 was considered to be statistically significant.

**Cell proliferation**

All cells were cultured at 37 °C in humidified atmosphere with 5% CO_2_. The number of proliferated cells was counted for 3 days after the cells (1 × 10^6^) were seeded in a 100 mm dish.

**Replicative life span**

IHOK-S and IHOK-P cells were routinely maintained in subconfluent conditions. The number of population doubling (PD) was determined using the following formula; PD = log (N/N_0_)/log 2, where N is the number of collected cells and N_0_ is the number of seeded cells.

**References**

1. Illeperuma RP, Park YJ, Kim JM, Bae JY, Che ZM, Son HK, et al. Immortalized gingival fibroblasts as a cytotoxicity test model for dental materials. J Mater Sci Mater Med. 2012;23: 753-762. doi:10.1007/s10856-011-4473-6 PMID:22071981

2. Lee EJ, Kim J, Lee SA, Kim EJ, Chun YC, Ryu MH, et al. Characterization of newly established oral cancer cell lines derived from six squamous cell carcinoma and two mucoepidermoid carcinoma cells. Exp Mol Med. 2005;37: 379-390. doi:10.1038/emm.2005.48 PMID:16264262

3. David CJ, Chen M, Assanah M, Canoll P, Manley JL. HnRNP proteins controlled by c-Myc deregulate pyruvate kinase mRNA splicing in cancer. Nature. 2010;463: 364-368. doi:10.1038/nature08697 PMID:20010808

4. Clower CV, Chatterjee D, Wang Z, Cantley LC, Vander Heiden MG, Krainer AR. The alternative splicing repressors hnRNP A1/A2 and PTB influence pyruvate kinase isoform expression and cell metabolism. Proc Natl Acad Sci U S A. 2010;107: 1894-1899. doi:10.1073/pnas.0914845107 PMID:20133837

5. Witton CJ, Hawe SJ, Cooke TG, Bartlett JM. Cyclooxygenase 2 (COX2) expression is associated with poor outcome in ER-negative, but not ER-positive, breast cancer. Histopathology. 2004;45: 47-54. doi:10.1111/j.1365-2559.2004.01898.x PMID:15228443
